# Non-invasive Assessment of Pulmonary Artery Wave Reflection in Dogs With Suspected Pulmonary Hypertension

**DOI:** 10.3389/fvets.2021.659194

**Published:** 2021-07-09

**Authors:** Tomohiko Yoshida, Katsuhiro Matsuura, Goya Seijirow, Akiko Uemura, Zeki Yilmaz, Ryou Tanaka

**Affiliations:** ^1^Department of Veterinary Surgery, Tokyo University of Agriculture and Technology, Fuchu-shi, Japan; ^2^Department of Bioresource Sciences, Nihon University, Fujisawa-shi, Japan; ^3^Department of Clinical Veterinary Medicine, Obihiro University of Agriculture and Veterinary Medicine, Obihiro-shi, Japan; ^4^Department of Internal Medicine, Uludag University, Bursa, Turkey

**Keywords:** wave intensity analysis, Doppler echocardiography, pulmonary hypertension, wave separation analysis, wave reflection

## Abstract

**Background:** Pulmonary arterial wave reflection (PAWR) occurs when the forward blood flow out the right ventricle is reflected by the pulmonary arterial tree, generating a backward wave. PAWR assessed by cardiac catheterization has been used to obtain information regarding pulmonary artery hemodynamics in pulmonary hypertension (PH) in people. However, diagnostic cardiac catheterization is not commonly used in small animal medicine because it is invasive and requires anesthesia.

**Hypothesis/Objective:** To investigate whether PAWR can be assessed non-invasively in dogs with suspected PH using Doppler echocardiography, based on wave intensity analysis (WIA). In addition, the method was validated in a dog model of acute pulmonary embolism.

**Animals:** Fifty-one client-owned dogs with tricuspid valve regurgitation were included in the clinical study (35 with suspected PH and 16 without echocardiographic evidence of PH) and eight healthy beagle dogs were included in the validation study.

**Methods:** PAWR was assessed by separating pulmonary artery pulse pressure waveforms, which were estimated from the flow profile of tricuspid regurgitation, into forward (Pf) and backward pressures (Pb) using WIA. Reflection coefficient (RC) was defined as the ratio of peak Pb to peak Pf. We investigated the relationships between RC, cause, and survival time in dogs with suspected PH. In addition, we performed a validation study to compare PAWR obtained by cardiac catheterization and PAWR by Doppler echocardiography in dogs with experimentally-induced PH.

**Results:** RC was significantly higher in dogs with suspected PH than in dogs without echocardiographic evidence of PH (0.18 ± 0.13 vs. 0.59 ± 0.21, *P* < 0.001). A characteristic reflected waveform appeared depending on the cause of PH. Kaplan-Meier survival curves showed that dogs with RC > 0.48 had a significantly shorter survival time than dogs with RC <0.48 (x^2^ = 9.8, log-rank test, *p* = *0.0018*, median survival time 353 days vs. 110 days). In the validation study, RC obtained by Doppler echocardiography was significantly correlated with RC obtained by cardiac catheterization (*r* = 0.81, *P* < 0.001).

**Conclusions:** PAWR analysis performed by echocardiography seems feasible in dogs and could provide useful information for classification and prognosis in canine PH.

## Introduction

Pulmonary hypertension (PH), defined in humans as a mean pulmonary arterial pressure (PAP) ≥ 25 mmHg at rest measured by right heart catheterization, is a pathological condition characterized by an increased pulmonary arterial pressure, and which can lead to right ventricular dysfunction (1–4). In small animals, right heart catheterization is not often performed as a diagnostic procedure because it is an invasive technique requiring general anesthesia, difficulting diagnosis and severity assessment of PH (5). Although the measurement of estimated pulmonary artery systolic pressure using tricuspid valve regurgitation (TR) is often used as a screening tool for the diagnosis and severity of PH, the accuracy of this method has been questioned because it is affected by right ventricular dysfunction and technical errors (6, 7). In human medicine, the direct measurement of PAP and pulmonary vascular resistance (PVR) and pulmonary vein wedge pressure represents the gold standard for PH diagnosis and characterization (4, 8). Recently, several studies have reported that pulmonary arterial wave reflection (PAWR) might provide additional information about right ventricular afterload (9, 10). PAWR occurs when the forward blood flow out the right ventricle is reflected by the pulmonary arterial tree, generating a backward wave. PAWR can be calculated by measuring pulmonary artery flow and pressure simultaneously using wave intensity analysis (WIA), as proposed by Parker and Jones (11–13). PAWR, determined by cardiac catheterization, has been used to obtain information regarding pulmonary artery hemodynamics in PH (10, 14).

The purpose of this study was to assess PAWR non-invasively using Doppler echocardiography in dogs with suspected PH. In addition, we performed a validation study to determine whether assessment by echocardiography correlates accurately with assessment by right heart catheterization (RHC) in dogs with experimentally induced PH.

## Materials and Methods

### Study Protocol

This study was prospectively conducted at two private veterinary hospitals and two universities in Japan between April 2018 and September 2020. Ethical approval was not required because we used the images taken during the routine examination. Echocardiographic measurements were performed by four veterinarians with expertise in the small animal cardiology field.

The non-invasive assessment of PAWR proposed in this study requires analysis of TR and RVOT spectral Doppler signals, and availability of a synchronous electrocardiogram. The calculation method is described in detail at the end of this section. A standardized protocol was used by the different investigators, for which the images obtained from the view allowing the clearest Doppler signal of TR were used, and the sweep speed of the synchronous electrocardiogram was set to 300 cm/s.

The study consisted of two parts: a clinical study and a validation study. Dogs were included in the clinical study if TR was identified by echocardiography. Next to echocardiography, all dogs needed to have undergone physical examination, thoracic radiography and blood testing (complete blood count, serum biochemistry).

The definitive diagnosis of PH requires RHC. However, RHC is difficult to apply in small animals, therefore, PH was suspected if a maximal TR velocity of ≥3.4 m/s was observed, based on Doppler echocardiographic assessment, and if the dogs presented with PH-related clinical signs, as outlined below. Furthermore, PH was suspected if a maximal TR velocity of ≥2.9 m/s was observed, based on Doppler echocardiographic assessment, and if other echocardiographic findings suggestive of PH were found in dogs presenting with PH-related clinical signs, as outlined below. Clinical signs considered possibly related to PH included: syncope without another identifiable cause, respiratory distress at rest, activity or exercise terminating in respiratory distress, abdominal distention due to ascites, tachypnea at rest, increased respiratory effort at rest, prolonged post-exercise or post-activity tachypnea, and cyanotic or pale mucous membranes (5). Other echocardiographic findings were defined as flattening of the interventricular septum (especially systolic flattening), pulmonary artery enlargement, systolic notching of the Doppler RV outflow profile (5). Dogs with TR < 2.9 m/s and no echocardiographic abnormality suggestive of PH or possibly related clinical signs were included in the control group. After dogs underwent radiographs and blood testing, blood gas analysis, computed tomography (CT) examination, and histopathological examination after autopsy were performed at the discretion of the attending clinician. Dogs were excluded from this study if they had suspected PH without measurable TR, if PH was deemed possibly secondary to a tumor, if a concomitant congenital heart disease (such as pulmonic stenosis, patent ductus arteriosus and ventricular septal defect) was present, and in case of arrhythmias other than sinus arrhythmia. The clinician recorded whether the case has syncope by questioning the owner. In addition, the clinician confirmed whether the case has edema (ascites, pleural effusion) due to right heart failure by ultrasonography. Clinical data, conventional echocardiographic indices, wave reflection indices, and survival times were collected for all cases.

### Survival Time and PH Cause Determination

Survival time was identified from the day of first visit to either the day of spontaneous death or the day of euthanasia. The end point of the study was death, and no censorship was performed in this study (all patients died within the observation period). The cases suspected with PH were categorized into six groups based on the PH consensus established by the ACVIM (5). Briefly, cases with suspected PH due to left heart disease were classified into group 2, and cases with suspected PH due to lung disease based on the thoracic radiography imaging results or CT scan were classified into group 3. Cases with suspected PH due to pulmonary thromboembolism, as confirmed by CT, echocardiography, or histopathological examination after autopsy, were classified into group 4. Cases with suspected PH secondary to heartworm disease, confirmed by antigen test positive and identification of an adult worm using echocardiography were classified into group 5. Other cases, for which no left atrial enlargement was identified, were classified into group 1. Cases with suspected PH associated with multiple factors were classified into group 6. For the purpose of this study, dogs in group 4 and 5 were united in a single category, defined as “thrombosis/heartworm disease class,” whereas dogs in groups 1, 2, 3 and 6 were united in another category, defined as “other class.”

### Conventional Echocardiography and Doppler Examination

Echocardiography was performed by a veterinarian with more than 5 years of clinical experience and with expertise in the cardiology field. Echocardiographic examinations were performed using a ProSound F75 Premier CV with a 5-MHz transducer (Hitachi Aloka Medical, Tokyo, Japan). TR velocity, obtained by continuous–wave Doppler, was measured from the view that allowed the clearest envelope of the TR velocity and maximum speed (5). The flattening of the interventricular septum was identified on M-mode images from the right parasternal short-axis view (15). The RVOT and main pulmonary artery-to-aortic root diameter ratio (MPA/AO) were measured from the standard right parasternal short-axis view (16, 17). RVOT flow was assessed with pulse-wave Doppler, and obtained placing the sample volume (2 mm) centrally between the opened pulmonary valve leaflets. Ejection time (ET), acceleration time (AcT), and AcT/ET ratio were assessed using RVOT flow profiles as follows. The AcT was measured as the time between the onset of the Doppler flow signal to the peak flow velocity. ET was measured from the onset of the Doppler RVOT signal to the end of the signal, and the AcT: ET ratio was calculated (16, 17). The end-diastolic MPA diameter was measured just below the closed pulmonary valve, the aortic diameter was measured from the same view, and the MPA/AO ratio was calculated.

### The Method of Measuring PAWR in Right Heart Catheterization

We performed a validation study to determine whether assessment of PAWR by Doppler echocardiography correlates accurately with assessment of PAWR by RHC (Reference to **Validation study on the non-invasive measurement of PAWR: A pilot study**). Before explaining validation study, section **The method of measuring PAWR in right heart catheterization** and section **The echo-Doppler method of assessing PAWR** describes the measurement method of PAWR by RHC and by Doppler echocardiography. The following is an explanation using the catheter method.

PAWR was generated when pulmonary artery blood flow is reflected from the peripheral pulmonary vascular wall and can be gained by separating pulmonary artery pulse pressure into forward (Pf) and backward pressures (Pb) using the concept of wave intensity analysis (WIA) (10, 11, 18, 19). The process of this analysis is started by calculating the wave speed (WS). A simple way to estimate WS, which represents the local elastic properties of the artery, is to measure the pulmonary artery pressure (P) and flow (U) simultaneously using a dual sensor-tipped pressure and flow wire (Combowire, Royal Philips, Amsterdam, Netherlands) and to plot instantaneous measurements of P vs. U. This wire can measure P and U simultaneously. Plotting the instantaneous measurements of P and U makes a P-U-loop. The WS is expressed as the slope of the P-U loop and can take advantage the water hammer equation relating P and U on the condition that there is no wave reflection in early systole:

(1) $$\begin{array}{l} c\;=\;\left(dP\;/\;dU\right)\;/\;\rho \end{array}$$

where, dP and dU are the changes in P and U, ρ is the density of blood (1,050 kg/m^3^) and c is WS. Pulse pressure can be separated into those attributed to forward-traveling (Pf) and backward-traveling (Pb) waves using equations 2 and 3 (20).

(2) $$\begin{array}{l} dPf\;=\;\left(dP\;+\;\rho c\;dU\right)\;/\;2 \end{array}$$

(3) $$\begin{array}{l} dPb=\;\left(dP-\;\rho c\;dU\right)\;/\;2 \end{array}$$

where, dPf is the temporal change in Pf, and dPb is the temporal change in Pb. Pf and Pb can then be determined by summing the differences.

(4) $$\begin{array}{l} Pf\;=\;\Sigma dPf \end{array}$$

(5) $$\begin{array}{l} Pb\;=\;\Sigma dPb \end{array}$$

**Figure 1:**
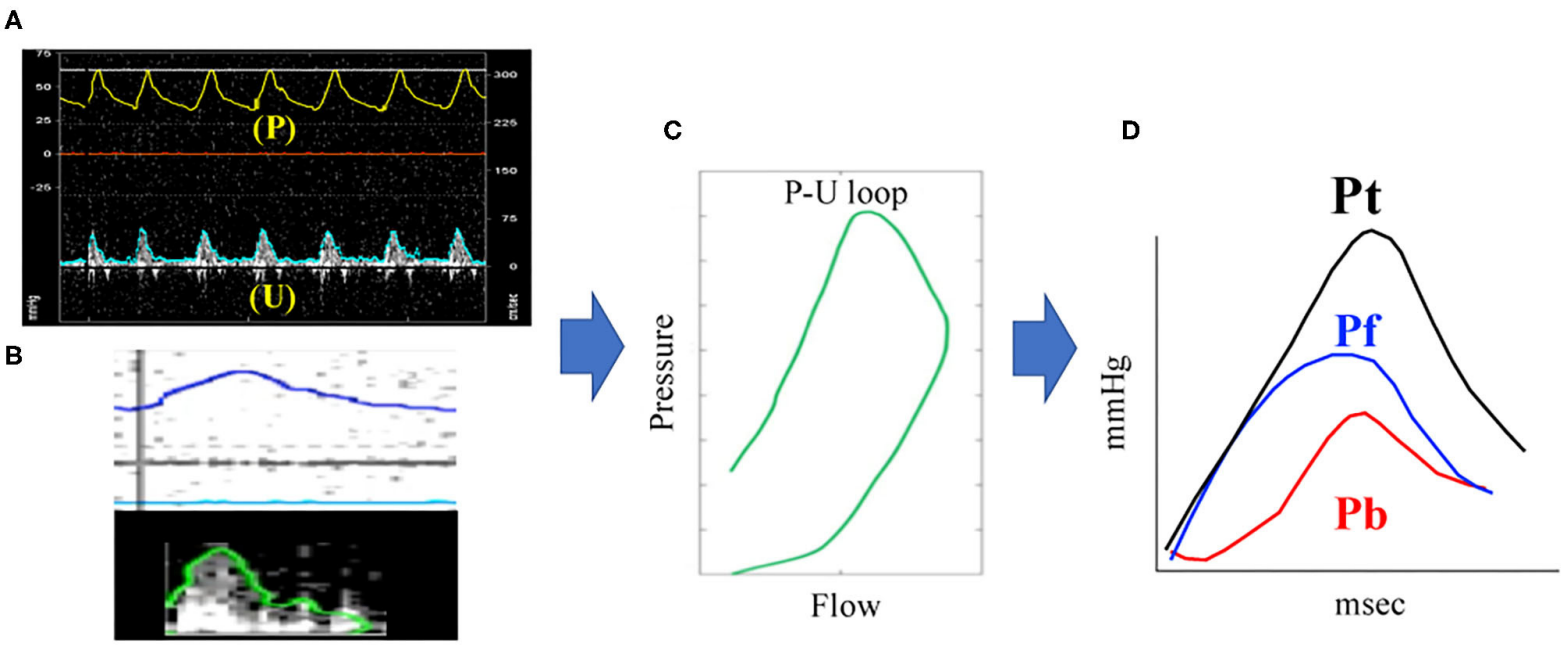
PAWR assessment by right heart catheterization. Process of **(A–D)** were processed using an in-house program code written in MATLAB. **(A)**, pulmonary artery pressure (P) and flow (U) gained by a dual sensor-tipped pressure and flow wire simultaneously. **(B)**, Contour detection of P and U. The P and U waveforms measured directly with the catheter were smoothed using a Savitzky–Golay filter and then ensemble-averaged over three cardiac cycles. **(C)**, Determination of the P-U loop. **(D)**, PAWR indices gained by wave separation analysis. PAWR, Pulmonary arterial wave reflection; Pt, Total pressure; Pf, Forward pressure; Pb, Backward pressure.

The obtained P and U were processed in MATLAB (MathWorks 2019b, Massachusetts, USA) using the above formula to calculate the PAWR (Figure 1). This analysis yields three wave reflection indices: peak Pb, peak Pf and reflection coefficient (RC) calculated as the ratio of peak Pb to peak Pf.

### The Echo-Doppler Method of Assessing PAWR

The non-invasive method we propose herein uses echo-Doppler derived Pressure (P) and flow (U), instead of the direct measurements. (P) and (U) were indirectly assessed as follows.

A pulsed-wave Doppler tracing of RVOT flow was used as a surrogate for U waveform. On the other hand, P waveform is estimated by applying the simplified Bernoulli equation to a continuous-wave Doppler tracing of TR flow and adding a term of right atrial pressure as below.

(6) $$\begin{array}{l} P\left(t\right)\;=\;4\;\times \;TRV\left(t\right)2\;+\;RAP \end{array}$$

where, t is time, TRV is TR velocity and RAP is right atrial pressure which we assume is constant throughout the cardiac cycle. End-diastolic P is determined as *P-*value at the beginning of ejection (shown as t0) identified from the U waveform.

(7) $$\begin{array}{l} End-diastolic\;P\;=\;4\;\times \;TRV\left(t0\right)2\;+\;RAP \end{array}$$

Subtracting end-diastolic P from P waveform yields pulse pressure waveform; this subtraction eliminates the term of right atrial pressure.

(8) $$\begin{array}{l} Pulse\;pressure\left(t\right)\;=\;4\;\times \;TRV\left(t\right)2\;-\;4\;\times \;TRV\left(t0\right)2 \end{array}$$

**Figure 2:**
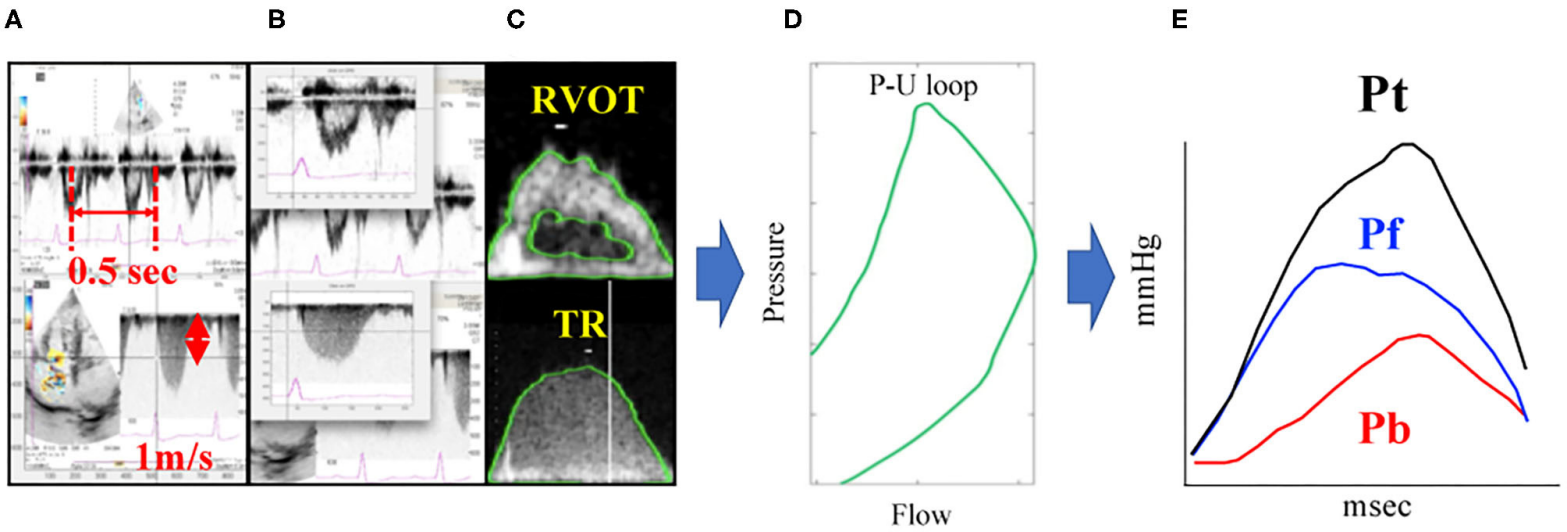
PAWR assessment by Doppler echocardiography. Process of **(A–E)** were processed using an in-house program code written in MATLAB. **(A)**, Defining the time axis and flow velocity axis. **(B)**, Synchronizing TR flow and RVOT flow from the electrocardiogram. **(C)**, Contour detection of TR flow and RVOT flow. The envelope of the RVOT flow Doppler signal was traced semi-automatically to obtain the U waveform. The P waveform was estimated by applying the simplified Bernoulli equation to a semi-automatic tracing of the envelope of the TR Doppler signal. These waveforms were smoothened using a Savitzky–Golay filter and then ensemble-averaged over three cardiac cycles with reference to the R wave on the electrocardiogram. **(D)**, Determination of the P-U loop. **(E)**, PAWR indices gained by wave separation analysis. This sample dog is same dog in Figure 1. TR, tricuspid regurgitation; RVOT, right ventricular outflow tract; PAWR, pulmonary arterial wave reflection; Pt, total pressure; Pf, forward pressure; Pb, backward pressure.

As described above, (P) is gained by Doppler echocardiography using equation from (6) to (8). Pf and Pb can be gained by applying (U) derived from RVOT waveform and (P) derived from TR waveform to (1)–(5) equations. Similar to the catheter method, Doppler method also yields three wave reflection indices: peak Pb, peak Pf and RC. The Doppler echocardiography method for the assessment of PAWR is shown in Figure 2. These calculations were also performed using MATLAB.

### Validation Study on the Non-Invasive Measurement of PAWR: A Pilot Study

This new non-invasive method was verified based on the results of a study in which we compared RC values determined by catheter with those derived by echo-Doppler. Eight healthy beagle dogs (Kitayama Labs, Nagano, Japan) were used in this study (all female, aged 4–5 years old, weighing 10–13 kg). This validation experiment was approved by the Animal Experimental Committee of the Tokyo University of Agriculture and Technology (Approval number: R03-33). All animal experiments were conducted in accordance with the regulations on animal experiments and the Guide for the Care and Use of Laboratory Animals of the Tokyo University of Agriculture and Technology.

To assess whether the new Doppler echocardiography method can be used to detect alterations in PAWR associated with the development of PH, each dog was administered repeated injections of dextran microspheres cross-linked with epichlorohydrin (Sephadex G-50, GE Healthcare, diameter 300 μm) into the pulmonary artery until the mean PAP increased above 30 mmHg. Measurements of RC by Doppler echocardiography and catheterization were performed both before and after epichlorohydrin injection under anesthesia. All dogs were sedated with Buprenorphine hydrochloride (Lepetan; Otsuka Pharmaceutical Co., Ltd., Tokyo, Japan, 0.02 mg/kg, intravenously), midazolam hydrochloride (Dormicum; Astellas Pharma Inc, Tokyo, Japan, 0.2 mg/kg, intravenously), Atropine sulfate (Atropine sulfate; Tanabe Seiyaku Co., Ltd., Saitama, Japan, 25 μg/kg, intravenously). Anesthesia was induced with propofol (Propofol Mylan; Mylan Seiyaku, Tokyo, Japan, 4 mg/kg, intravenously) after tracheal intubation and anesthesia was maintained by isoflurane inhalation (Isoflurane for Animal Use; Intervet, Osaka, Japan, end-tidal concentration of 1.5 ± 0.1%). A dual sensor-tipped pressure and flow wire was advanced to approximately 1 cm beyond the pulmonary valve through a 4.2-Fr multipurpose angiographic catheter (Goodtec angiographic catheter, GOODMAN, Aichi, Japan) inserted from the left jugular vein to obtain the P and U waveforms. PVR was measured as [mean PAP-mean left atrial pressure (LAP)]/cardiac output. LAP and Right atrial pressure (RAP) were obtained from another 4.2-Fr multipurpose angiographic catheter inserted into the left atrium and right atrium through the left carotid artery and left jugular vein (The catheter placed in the left atrium and right atrium were also used to sample blood to calculate cardiac output). Cardiac output was calculated using the Fick method from oxygen consumption estimated using Sykes' formula (21).

### Statistical Analysis

Continuous data are expressed as the mean ± standard deviation (SD). Categorical data are expressed as numbers and percentages. The level of significance was set at *p* < 0.05.

**Figure 3:**
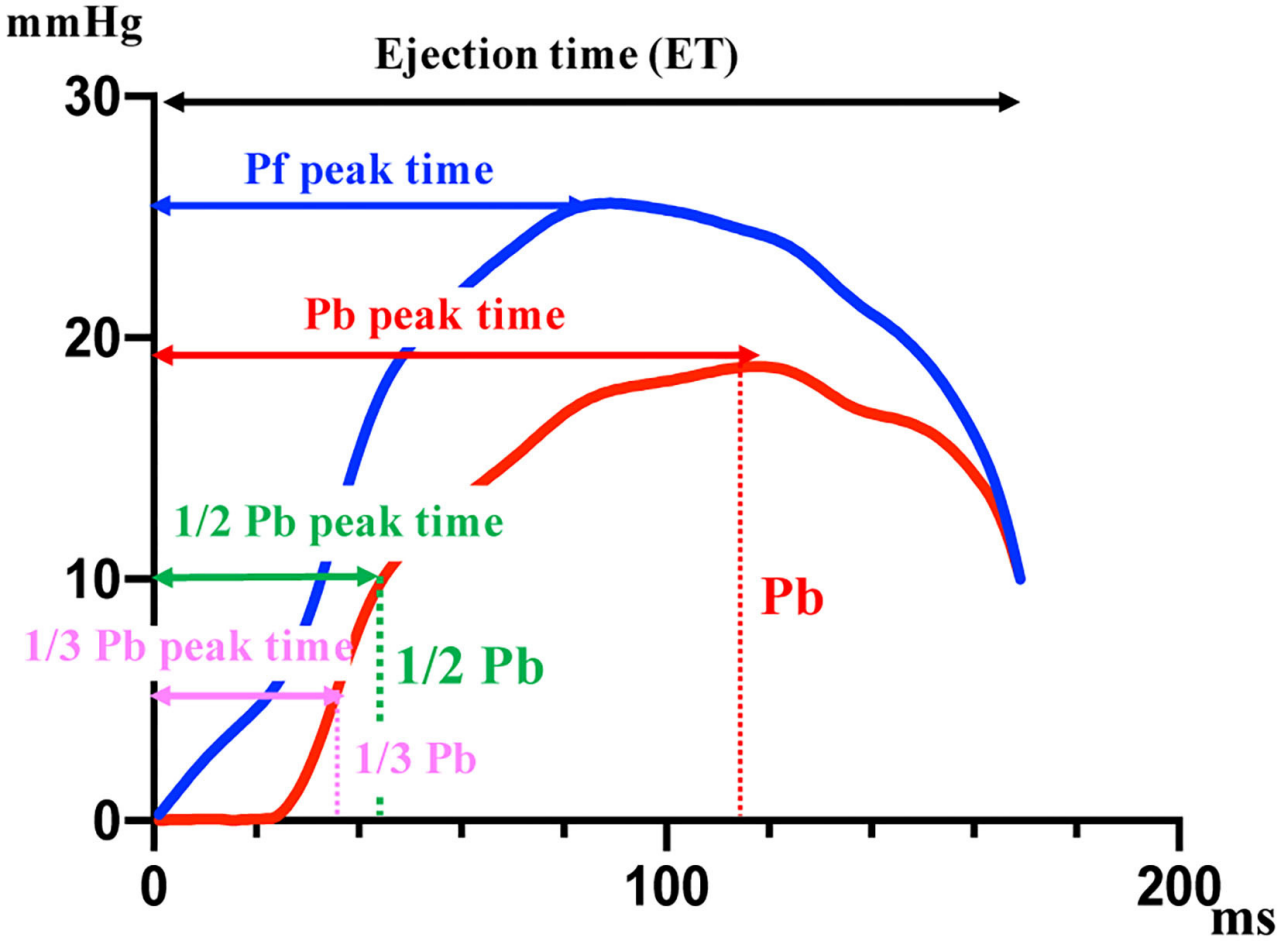
The variables of time phase in PAWR. The variables shown in this figure are associated with the difference between the PH other class and PH thrombosis/heartworm disease class. Pf, forward pressure; Pb, backward pressure. This figure shows the variables of time phase in PAWR of patient with thrombosis in the pulmonary artery.

The normal distribution of the data was evaluated using the Kolmogorov–Smirnov test, and the assumption of homogeneity of variances was determined using Bartlett's test. For normally distributed parameters, differences between the groups were analyzed using a paired *t*-test. For non-parametric parameters, differences between groups were analyzed using the Mann-Whitney *U* test. For normally distributed parameters, differences between three groups were analyzed using a one-way ANOVA followed by *post hoc* analysis with Bonferroni correction for normally distributed parameters. For non-parametric parameters, differences between groups were evaluated using a non-parametric Kruskal-Wallis test followed by *post hoc* analysis with Dunn's multiple comparison test. Spearman's correlation analysis was used to examine the correlation between RC evaluated by Doppler and RC evaluated using a catheter. A Bland-Altman plot was used to test for random and systematic errors between the RC by Doppler and RC by catheter. Cut-off values were based on significantly different sequential quartiles, which were used to dichotomize each variable. Cut-offs were calculated using receiver-operating characteristic (ROC) curve analyses and dot plots. Positive and negative predictive values were calculated for each cut-off value. The area under the ROC curve (AUC) and 95% confidence interval (CI) were calculated for each variable. Optimal cut-off values were chosen for each variable based on the highest Youden index. Kaplan-Meier curves were constructed, and log-rank analyses were performed to assess the effects of the cut-off values on survival time. The variables of time phase in PAWR was analyzed by multivariate discriminant analysis to investigate the factors associated with the difference between the PH other class and PH thrombosis/heartworm disease class. The variables of time phase in PAWR were defined as Peak of Pb (Pb peak), time to reach peak of Pb (Pb peak time), Pb peak time divided by ejection time (Pb peak time/ejection time), time to reach 1/2 peak of Pb divided by ejection time (1/2 Pb peak time/ejection time), time to reach 1/3 peak of Pb divided by ejection time (1/3 Pb peak time/ejection time), and difference between Pb peak time and Pf peak time divided by the ejection time [(Pb peak time–Pf peak time)/ejection time] (Figure 3). Discriminant analysis was performed using Pb peak, Pb peak time, Pb peak time/ejection time, 1/2 Pb peak time/ejection time, 1/3 Pb peak time/ejection time, and (Pb peak time–Pf peak time)/ejection time, as independent variables.

Statistical analyses were performed using SPSS (Statistical Package for Social Science; International Business Machines Corporation, Chicago, USA).

## Results

### Study Group

PH was suspected in 35 of 51 patients with TR. These included 24 cases assigned to the category of dogs with no definitive diagnosis (four cases were suspected PH of group 1. Eleven cases were suspected PH of group 2. Nine cases were suspected PH of group 3. Eleven cases were classified as thrombosis/heartworm disease class five cases were suspected as PH of group 4. Six cases were suspected as PH of group 5). Table 1 shows the characteristics of the patients and the echocardiographic and wave reflection indices measured in this study. The 71.4% of patients with suspected PH have syncope. The 25.7% of patients with suspected PH has ascites and/or pleural effusion. The MPA/AO of dogs with suspected PH was significantly higher than that of dogs without PH (*P* < 0.001). The AcT and AcT/ET values of dogs with suspected PH were significantly lower than those of dogs without PH (*P* < 0.001). In addition, 45.7% of PH cases presented with septal flattening, and 22.9% had a notch in the pulmonary artery waveform. The Pf, Pb, and RC values of cases with suspected PH, as determined by WIA, was significantly higher compared to those of cases without PH (*P* < 0.001).

**Table 1:** Baseline clinical data and echocardiographic variables.

	**Control**	**PH**
**Baseline clinical data**		
n	16	35
Age	12 ± 2.0	13 ± 2.5
male (%)	73	63
Total clinical score	1 ± 1	5.5 ± 2.8^*^
Body weight (kg)	4.6 ± 2.9	6.6 ± 3.3
Syncope (%)	0	71.4
Ascites and/or pleural effusion (%)	0	25.7
**Echocardiographic indices**		
MPA/AO	0.8 ± 0.1	1.1 ± 0.2^*^
RVOT flow		
Peak velocity, cm/s	78.5 ± 15.9	80.3 ± 22.3
AcT, ms	71 ± 13.4	63.8 ± 17.9^*^
AcT/ET	0.41 ± 0.09	0.32 ± 0.08^*^
TR flow peak velocity, cm/s	276 ± 61	406 ± 70^*^
Septal flattening: number (%)	0	45.7
Notched RVOT flow: number (%)	0	22.9
**Wave reflection analysis**		
Forward pressure (Pf)	24 ± 8.9	37.2 ± 11.9^*^
Backward pressure (Pb)	3.9 ± 2.9	21.9 ± 9.1^*^
Reflection coefficient (RC: Pb/Pf)	0.18 ± 0.13	0.59 ± 0.21^*^

*PH, Pulmonary Hypertension; MPA/AO, main pulmonary artery/aorta; RVOT, right ventricular outflow tract; AcT, acceleration time; ET, ejection time; TR, tricuspid regurgitation*.

* *p < 0.05, vs. control*.

### Characteristics of PAWR in Each Group

**Figure 4:**
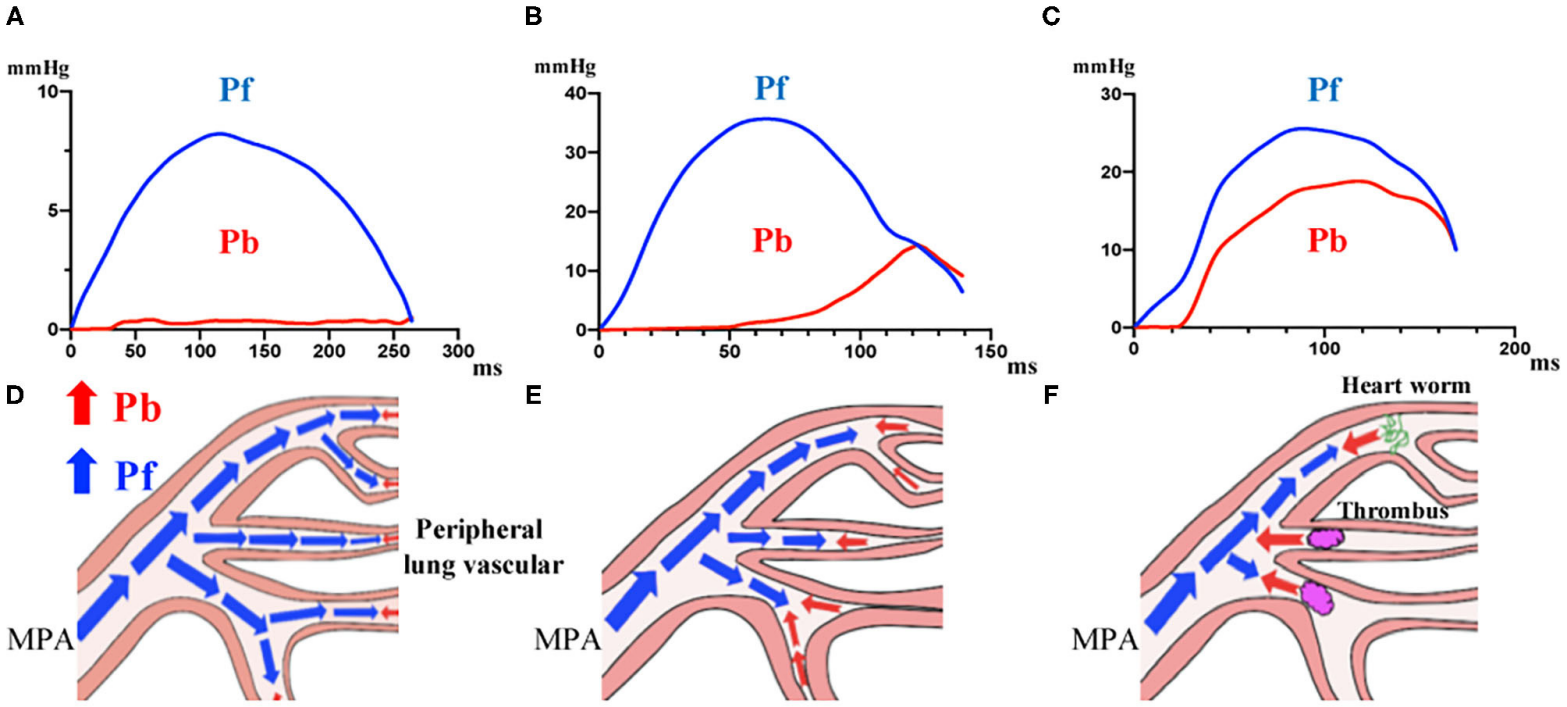
Waveforms in dogs grouped in different categories. **(A)**, Dogs without echocardiographic evidence of PH. **(B)**, Dogs with suspected PH and no definitive diagnosis. **(C)**, Dogs with suspected PH due to thrombosis/heartworm disease. **(D–F)**, Schema representing the place where wave reflection appears in each group. PH, Pulmonary Hypertension.

While comparing PAWR between categories, a difference was observed concerning the waveforms, as shown in Figure 4 and explained hereafter. In the group of dogs without echocardiographic evidence of PH, the P waveform was primarily composed of Pf (Figure 4A). In dogs with suspected PH, the Pf peak, Pb peak and RC were significantly higher than in the former group, and the time when Pb was generated and the peak time of Pb were different between dogs in the thrombosis/heartworm disease class and dogs in the other class. In the waveforms of the other PH classes, the Pb peak shifted to the right (Figure 4B). In contrast, Pb was generated earlier in the PH thrombosis/heartworm disease class than in the PH other class, and Pb peaked earlier in the PH thrombosis/heartworm disease class than in the PH other class (Figure 4C). Figures 4D–F show schemas that predict where reflections appear.

**Figure 5:**
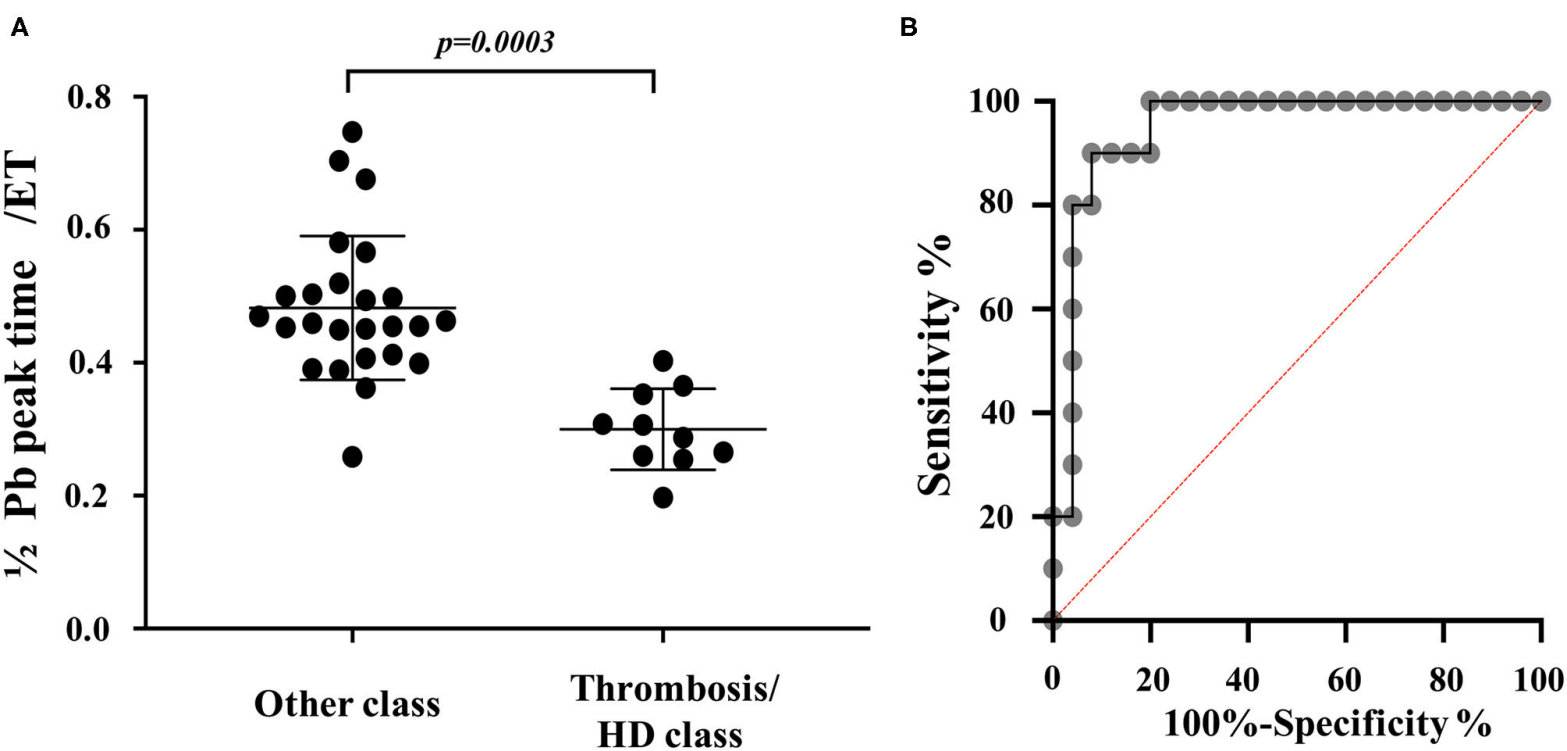
PAWR indices in dogs grouped in different categories. **(A)**, Comparison of 1/2 Pb peak time/ejection time between dogs with suspected PH and no definitive diagnosis and Dogs with suspected PH due to thrombosis/heartworm disease. **(B)**, Receiver-operating characteristic (ROC) curves of 1/2 Pb peak time/ejection time for distinguishing between dogs with suspected PH and no definitive diagnosis and Dogs with suspected PH due to thrombosis/heartworm disease among dogs with PH. PH other class, dogs with suspected PH and no definitive diagnosis. PH thrombosis/HD class, PH due to thrombosis/heartworm disease. PH, pulmonary hypertension; Pb, backward pressure (Pulmonary artery wave reflection); ET, ejection time; HD, heartworm disease.

Discriminant analysis determined the variables that most affect the difference in waveform between the other classes and thrombosis/heartworm disease classes. From the discriminant analysis, 1/2 Pb peak time/ejection time had the greatest effect on the waveform differences between other classes and the thrombosis/heartworm disease classes (Table 2). We can explain the independent variables that were used in the discriminant analysis from Figure 3. Figure 5A shows the results of comparing 1/2 Pb peak time/ejection time between other classes and thrombosis/heartworm disease classes. 1/2 Pb peak time/ejection time of the PH thrombosis/heartworm disease class was significantly lower than that of the PH other class. ROC analysis revealed that the 1/2 Pb peak time/ejection time was able to classify an animal between the PH group and the thrombosis/heartworm disease class (Figure 5B; AUC: 0.95; cut-off value: 0.37; *P* < 0.001; sensitivity: 90%; specificity: 92%; 95% Cl: 58.1–84.3%; Youden index: 0.82).

**Table 2:** Coefficients of discriminant analysis associated with the difference between the PH other class and PH thrombosis/heartworm disease class.

	**Pb peak time**	**Pb peak time/ET**	**1/2 Pb peak time/ ET**	**1/3 Pb peak time/ET**	**(Pb peak time–Pf peak time)/ET**
Discrimination coefficient	−0.33	0.26	−1.722	0.96	−1.23

*Pb, backward pressure; ET, ejection time*.

### Relationship Between PAWR and Prognosis

**Figure 6:**
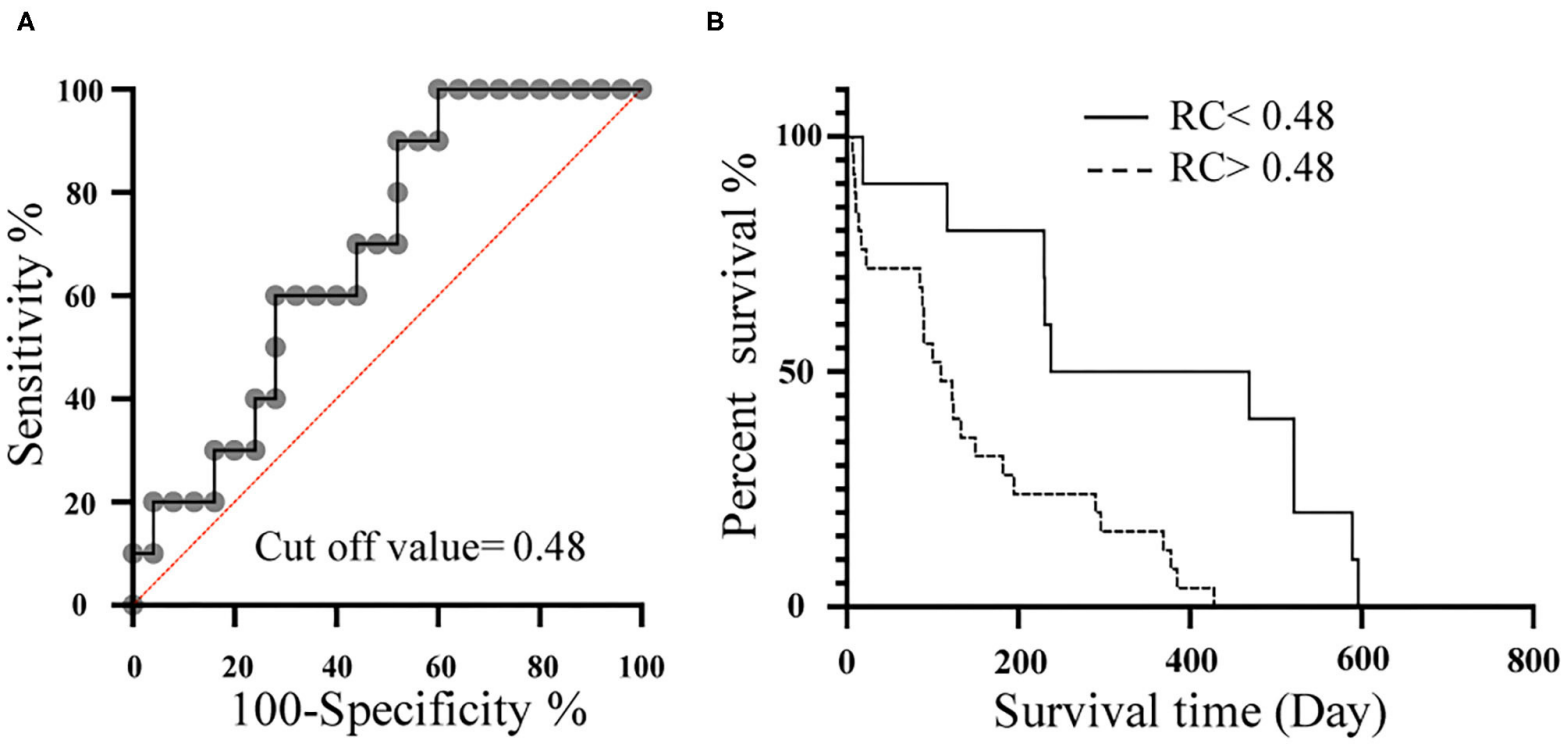
Relationship between RC and prognosis. **(A)**, Receiver-operating characteristic (ROC) curves of RC for the detection of dogs with edema related with right heart failure (ascites, pleural effusion) among dogs with PH. **(B)**, Kaplan-Meier survival curve for 35 dogs stratified by RC. Dotted line represents RC > 0.48. Solid line represents RC <0.48. RC, Reflection coefficient which calculated as the ratio of peak Pb to peak Pf; Pb, Backward pressure; Pf, Forward pressure.

We performed ROC analysis of cases with and without edema related with right heart failure (pleural effusion, ascites) to determine the cut off value for RC prognosis. The most accurate RC cut-off value for the identification of cases with edema related with right heart failure (ascites, pleural effusion) was 0.48 (Figure 6A; AUC: 0.81; *P* = 0.0026; sensitivity: 100%; specificity: 61%; 95% Cl: 69–92%; Youden index: 0.61). The Kaplan-Meier survival curves, when grouped according to the optimal RC cut-off values, demonstrated that patients with RC > 0.48, had a significantly shorter survival time than patients with RC <0.48 (Figure 6B; x^2^ = 9.8, log-rank test, *P* = 0.0018, median survival time 353 days vs. 110 days).

### Result of the Validation Study

**Figure 7:**
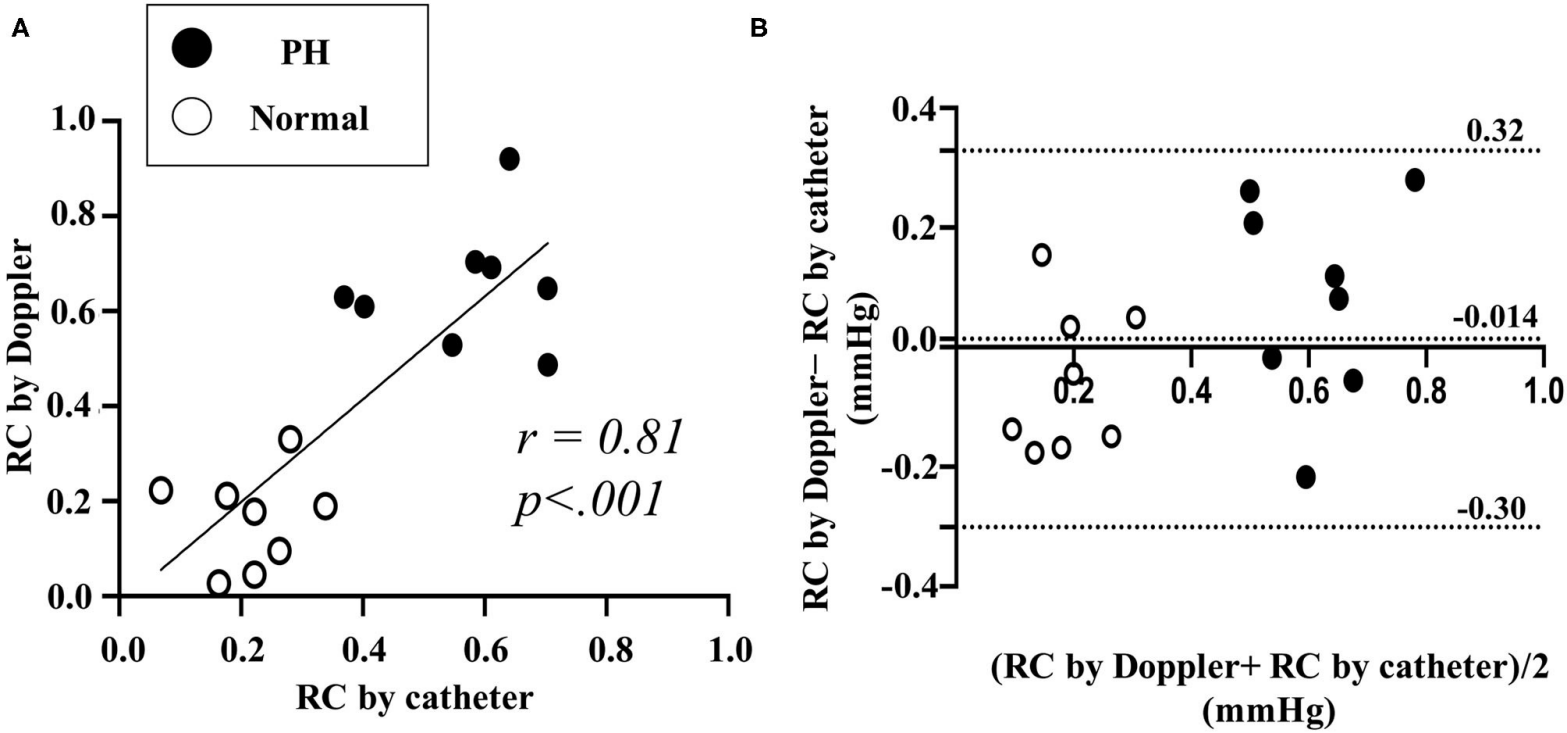
Comparison between RC by catheter and by Doppler in validation study using experimental Dog (Representative original imaging). **(A)**, Linear correlation between RC by the catheter and by the Doppler. **(B)**, Bland-Altman analysis for RC by catheter and by Doppler. RC, reflection coefficient which calculated as the ratio of peak Pb to peak Pf. Black dot represents the result of RC in pulmonary hypertension (PH). White dot represents the result of RC in normal condition.

RC calculated by Doppler echocardiography was found to be linearly correlated with RC evaluated by catheter (Figure 7A, RC by Doppler vs. RC by catheter: *r* = 0.81, *P* < 0.001). Figure 7B also shows the result of a Bland-Altman analysis between the RC values evaluated by the catheter and Doppler methods. The Bland-Altman analysis showed that the mean bias for differences in RC evaluated by Doppler compared with RC evaluated by catheter was −0.014 (SD: 0.16, 95% confidence interval, −0.30 0.32. The 95% of the data points were within ± 2 SDs of the mean difference. RC by Doppler significantly correlated with mean PAP and PVR (RC by Doppler vs. mean PAP: *r* = 0.88, *P* < 0.0001; RC by Doppler vs. PVR: *r* = 0.93, *P* < 0.0001).

## Discussion

This study investigated PAWR, as assessed by Doppler echocardiography measurements, in patients with suspected PH. Our findings suggest that PAWR can be estimated non-invasively using Doppler echocardiography and be a useful parameter possibly related to cause and prognosis in dogs with suspected PH.

PAWR can potentially provide novel information regarding pulmonary hemodynamics to supplement traditional methods used to evaluate PH, such as PAP and PVR, which are the most common hemodynamic measurements used to evaluate its progression (8–10, 14, 22, 23). In veterinary medicine, these parameters are not typically obtained invasively using cardiac catheterization (5). Therefore, in this study, we proposed that PAWR could be non-invasively obtained by Doppler echocardiography and attempted to measure PAWR non-invasively in clinical case.

Some paper reported that wave reflection can be measured non-invasively using image inspection. Quail et al. have demonstrated that non-invasive WIA could be performed using MRI in the branch pulmonary artery of healthy controls and patients with pulmonary arterial hypertension (PAH) and chronic thromboembolic pulmonary hypertension (CTEPH) (24). In our validation study using dogs with experimentally-induced PH, PAWR could be non-invasively obtained by Doppler echocardiography and that it was correlated with PAWR measured using cardiac catheterization. In addition, PAWR may be a hemodynamic parameter related to right ventricular afterload due to its significant correlation with PVR, which is a conventional index of right ventricular afterload.

PAWR parameter was significantly higher in patients with suspected PH. Wave reflection is generated when the vascular system, particularly vascular impedance, change between the proximal and distal vasculature due to vascular remodeling, arteriosclerosis, or thrombus (10, 25, 26). As PH develops, the pulmonary artery becomes a high-pressure, high-resistance, and low-compliance system (9, 10, 24). In PH patients, Pf is increased to maintain cardiac output in the face of increased pulmonary artery afterload, and Pb is also increased due to differences in the vascular impedance between the proximal and distal vascular system (10). Patients suspected with PH were significantly higher Pf, Pb, and RC values compared to those of the control group in this study. Furthermore, the PAWR measurements in suspected PH patients were associated with different waveforms depending on the cause of PH. Discrepancies in PAWR variables could be related to differences in the location at which level the reflection wave is generated. Castelain et al. reported that PAWR measurements associated with PAH and CTEPH differed. The PAP upstroke, systolic point of curvature, and reflected pressure wave appeared earlier in patients with CTEPH than in those with PAH (25). In this study, the 1/2 Pb peak time/ET was significantly lower in the PH other class than in the PH heartworm disease class, indicating that Pb was generated earlier during systole in PH patients with thrombosis than in patients without thrombosis. The presence of obstructions, such as thrombus or parasites, may change the reflecting site to a more proximal location. The results of this study show that indirect analysis of PAWR can be used as a non-invasive parameter to determine PH classification.

Furthermore, Kaplan-Meier survival analysis suggested that RC values were associated with the prognosis of suspected PH cases. RC is a better prognostic parameter for PH than Pb because Pb is a wave pressure that is generated after the forward wave conflicts with the peripheral vascular wall and is dependent on the magnitude of Pf. As PH advances and cardiac output decreases, Pf decreases, and the magnitude of Pb may also be reduced. Patients with severe PH who have right ventricular dysfunction have decreased Pb levels, which may underestimate the condition (10, 25). In contrast, RC may be a useful new evaluation method for PH that considers the ventricular-arterial coupling because RC represents the ratio between Pf, which represents right ventricular function, and Pb, which represents right ventricular afterload.

Because right ventricular catheterization, CT, and MRI cannot be easily performed without the use of anesthesia in small animals, severity evaluations are typically performed by observing symptoms and estimating systolic PAP (estimated from TR flow velocity) (5, 27, 28). In addition, classifying PH can be difficult, and treatment is often started without a clear cause. This study suggested that PAWR may contribute to distinguish between the PH other class and PH thrombosis/heartworm disease class and predict prognosis of PH. PAWR provides novel information about assessing pulmonary vascular disease such as a PH and is expected to contribute to patients in the near future.

In conclusion, PAWR indices was significantly higher in patients with suspected PH than in those without PH in our study. Furthermore, PAWR measurements are associated with the prognosis of PH cases, indicating that PAWR may affect the progression of PH. In addition, analysis of PAWR may also help to determine the classification of PH. Many factors that affect the pathophysiology of PH remain unclear, and conventional parameters do not always properly evaluate the severity of PH. Wave reflection, as determined by WIA, may provide additional information about assessing hemodynamics in pulmonary. Our study may facilitate the future use of PAWR in clinical settings because wave reflection can easily be obtained from Doppler echocardiography.

## Limitations

Because the scale of verification in this study was small, additional studies should be performed. For example, it is necessary to assess whether the new Doppler echocardiography method used in this study can detect alterations in the PAWR. The alterations in PAWR will be performed by administering an infusion load or a cardiac stimulant (PAWR may change as the cardiac output increases). We plan to perform additional study in the near future.

There are various causes of PH, and the model in this validation study has a hemodynamics similar to acute pulmonary artery embolism. Since the tendency of PAWR may differ depending on the cause, it is necessary to fully consider the results of this experiment that used model animals.

In clinical cases, measuring the PAWR in cases that do not exhibit TR can be difficult, as can the evaluation of wave reflection in cases that present with severe arrhythmia.

In this study, the number of PH cases was small; therefore, the difference in waveforms between groups should be analyzed further. The measurement of the PAWR can also have errors because the TR velocity may be underestimated or overestimated.

## Data Availability Statement

The raw data supporting the conclusions of this article will be made available by the authors, without undue reservation.

## Ethics Statement

The animal study was reviewed and approved by Animal Experimental subcommittee of Tokyo University of Agriculture and Technology (approval number: 30-146). Written informed consent was obtained from the owners for the participation of their animals in this study.

## Author Contributions

TY designed the study and wrote the initial draft of the manuscript. KM and GS acquired and analyzed the data. AU and ZY interpreted the results and critically reviewed the results. RT edited the manuscript and approved the final version of the manuscript. All authors contributed to the article and approved the submitted version.

## Conflict of Interest

The authors declare that the research was conducted in the absence of any commercial or financial relationships that could be construed as a potential conflict of interest.

## Abbreviations

AcT: acceleration time
ACVIM: American college of veterinary internal medicine
AO: aortic
AUC: area under the ROC curve
CI: confidence interval
CT: computed tomography
CTEPH: chronic thromboembolic pulmonary hypertension
ET: ejection time
MPA: main pulmonary artery
MRI: magnetic resonance imaging
MRI: magnetic resonance imaging
P: pressure
PAH: pulmonary Artery hypertension
PAP: pulmonary artery pressure
PAWR: pulmonary arterial wave reflection
Pb: backward pressure
Pf: forward pressure
PH: pulmonary hypertension
PVR: pulmonary vascular resistance
RC: reflection coefficient
ROC: receiver-operating characteristic
RVOT: right ventricular outflow tract
SD: standard deviation
TR: tricuspid valve regurgitation
U: velocity
WRI: wave reflection indices
PAWR: pulmonary arterial wave reflection.
